# Incidence and risk factors of exacerbations among COPD patients in primary health care: APMPOC study

**DOI:** 10.1186/1471-2458-9-8

**Published:** 2009-01-09

**Authors:** Eulàlia Borrell, Mar Rodríguez, Pere Torán, Laura Muñoz, Guillem Pera, Núria Montellà, Mònica Monteagudo, Magalí Urrea, Yolanda Puigfel, Antonio Negrete, Xavier Mezquiriz, Cristina Domènech, Anna Lacasta, Ma Llum García, Sandra Maneus, Glòria Tintoré

**Affiliations:** 1 Primary Healthcare Centre Sant Roc, Catalan Health Institute, Velez Rubio s/n, 08913 Badalona, Spain; 2 Primary Healtcare Centre Canet de Mar, Catalan Health Institute, Costa de l'Hospital s/n, 08640 Canet de Mar, Spain; 3 Primary Healthcare Research Support Unit Barcelonès Nord i Maresme. IDIAP Jordi Gol, Camí del Mig 36 (3^a ^planta), 08303 Mataró, Spain; 4 Primary Healthcare Methodology, Quality and Evaluation Unit Badalona i Sant Adrià de Besòs, Catalan Health Institute, Plaça de la Medicina, s/n, 08911 Badalona, Spain; 5 Primary Healthcare Research Support, IDIAP Jordi Gol. Avda, Gran Via de les Corts Catalanes 487, 08007 Barcelona, Spain; 6 Primary Healthcare Centre Gatassa, Catalan Health Institute, Camí del Mig 36 (4^a ^planta), 08303 Mataró, Spain; 7 Primary Healthcare Centre Llefià, Catalan Health Institute, Carretera Antiga de València s/n, 08913 Badalona, Spain

## Abstract

**Background:**

Worldwide, chronic obstructive pulmonary disease (COPD) is the fourth cause of death. Exacerbations have a negative impact on the prognosis of COPD and the frequency and severity of these episodes are associated with a higher patient mortality. Exacerbations are the first cause of decompensation, hospital admission and death in COPD. The incidence of exacerbations has mainly been estimated in populations of patients with moderate-severe COPD requiring hospital care. However, little is known regarding the epidemiology of exacerbations in patients with less severe COPD forms. It is therefore possible that a high number of these less severe forms of exacerbations are underdiagnosed and may, in the long-term, have certain prognostic importance for the COPD evolution. The aim of this study was to know the incidence and risk factors associated with exacerbations in patients with COPD in primary care.

**Methods and design:**

A prospective, observational, 3-phase, multicentre study will be performed involving: baseline evaluation, follow up and final evaluation. A total of 685 smokers or ex-smokers from 40 to 80 years of age with COPD, without acute respiratory disease or any other long-term respiratory disease will be randomly selected among the population assigned to 21 primary care centres. The diagnosis of COPD and its severity will be confirmed by spirometry. Information regarding the baseline situation, quality of life and exposure to contaminants or other factors potentially related to exacerbations will be collected. A group of 354 patients with confirmed COPD of varying severity will be followed for one year through monthly telephone calls and daily reporting of symptoms with the aim of detecting all the exacerbations which occur. These patients will be evaluated again at the end of the study and the incidence of exacerbations and associated relative risks will be estimated by negative binomial regression.

**Discussion:**

The results will be relevant to provide knowledge about natural history of the initial phases of the COPD and the impact and incidence of the exacerbations on the patients with mild-moderate forms of the disease. These data may be important to know the milder forms of exacerbation wich are often silent or very little expressed clinically.

## Background

COPD is characterized by little reversible airflow obstruction of chronic evolution and progressively incapacity causing a high morbimortality. Although it is a potentially avoidable disease, the rise in the prevalence of COPD is slow and unyielding, with epidemiological projections foreseeing a growing trend in the next decades and a two-fold increase in death by this disease in the next 20 years [1].

In Spain COPD is the fifth cause of death. The IBERPOC study estimated a prevalence of 9% among the Spanish population from 40 to 69 years of age and of 23% among the population of more than 60 years old [2]. This same study detected that underdiagnosis is high (78.2%) and that, in addition, it is accompanied by a worrisome undertreatment (80.7%). It is estimated that COPD makes up 2% of the Spanish healthcare budget representing approximately 0.25% of the gross national product. A recent study quantified the mean annual cost per patient diagnosed with this disease as $ 1,760 [3].

Chronic obstructive pulmonary disease may be diagnosed based on the clinical history, physical examination and the data provided by spirometry in primary healthcare. It has been demonstrated that the quality of the spirometries performed in primary care centres is adequate and allows effective diagnostic orientation [4]. The proximity of primary care to the patients also allows the disease to be approached in the early stages and is where most of the cases of COPD are attended.

Exacerbations are an important cause of the morbimortality of these patients, in whom the progressive course of COPD is often aggravated as a consequence of these episodes [5]. Exacerbations are the first cause of decompensation, hospital admission and death in COPD which, particularly in winter, lead to an overload in patients attended in hospital emergency departments and occupation of ward beds [6,7]. Moreover, a deterioration in pulmonary function may be observed, mainly following bacterial exacerbations [8], as well as in the short and long-term quality of life [9,10].

In a study performed in Spain in 2,414 outpatients diagnosed with chronic bronchitis and follow up of one month after the exacerbation, recurrence was reported in up to 21% of the patients. Of these, 32% required hospital attendance, 16% hospital admission and 51% were treated in primary care but required adjustment in their medication [8]. Considering the high prevalence of these diseases in the population, together with this rate of recurrences, which are initially evaluated in primary care, it may be inferred that this is a health problem with a high impact of the health status of these patients as well as in the use of healthcare services. Advances in the knowledge of the factors which condition the appearance of exacerbations and recurrence would be helpful to adopt preventive measures and to plan healthcare measures.

Over time different definitions of exacerbations have been proposed and used which, together with the use of different criteria of selection and indexes of patient severity, have made it difficult to compare the studies published. In 1999 the first consensus was proposed and was later ratified in 2004 [11]. This definition was based on three axes: the worsening of the conditions of the patients (without specifying which), acute onset and the need for changes in the normal treatment of the patient. Later the European Respiratory Society (ERS) and the American Thorax Society (ATS) made a joint consensus definition which considers an exacerbation of COPD to be an event in the natural development of the disease characterised by a change in baseline dyspnoea, cough and/or expectoration of the patients beyond the daily variations in the symptoms and which is sufficient to justify a change in treatment [12].

It is difficult to identify exacerbations based on their clinical definitions, thus, a wide variability may be observed in the different studies in relation to the concept of an exacerbation [13]. The patients themselves do not associate the worsening of their disease with the term exacerbation, although they are capable of consistently recognising the signs and symptoms which define or announce an exacerbation [14]. Identification of exacerbations is often based on the episodes referred by the patient, depending on their degree of memory. In a study by Meek [15], the concordance between the experience of COPD symptoms lived and the memory of the same two weeks later was acceptable, being influenced by the presence and intensity of the symptoms the day remembered and by the presence and grade of cognitive deterioration of the patient.

The incidence of exacerbations of COPD in Spain has recently been estimated in longitudinal studies carried out in outpatients with moderate-severe COPD, demonstrating a range of 1.5 – 2 episodes per year [10]. A high percentage of these episodes require medical attention and condition a high prescription of antibiotics. In studies undertaken in other countries, the incidence of exacerbations in moderate-severe COPD has been estimated to be 2.5 – 3 episodes per patient and year [16]. Nonetheless, it should be taken into account that a proportion of exacerbations do not receive medical care, with there being a certain degree of underdiagnosis [17] even in hospitalised patients with respiratory symptoms compatible with COPD [18]. Undertreatment may represent an accelerated decline in pulmonary function making early diagnosis and treatment essential [19].

The frequency of the exacerbations has been related to a greater degree of severity or deterioration in forced expiratory volume in the first second (FEV1) and to the season of the year [10]. Recurrences of exacerbations treated in the outpatient clinic have been related to the severity of COPD and the number of visits to the primary care physician in the last year. However, the initial severity of the exacerbation is not related to the recurrence [8]. It has been described that 50–70% of the exacerbations are due to infections, although the role of antibiotics in the treatment of an exacerbation and recurrence is controversial [20]. In a study performed in Spain [21] a relationship was found between the exacerbations and being a smoker, low compliance with inhalation therapy and severe involvement of pulmonary function. Common colds are associated with a greater severity and duration of exacerbations and have also been found to be important predictors of the appearance of an exacerbation. Other factors reported in the literature which may favour the appearance of exacerbations are environmental pollution, low temperatures and concomitant heart disease [8,22].

A two-year follow up study of patients with COPD demonstrated a significant worsening in the quality of life of patients who had exacerbations compared with those who did not, with this deterioration being greater in patients with two or more exacerbations. The impact of exacerbations on the quality of life was more important in the patients with better preserved pulmonary function [23].

There may be an important grade of underdiagnosis of exacerbations [16], particularly in the cases of mild-moderate COPD. The viral aetiology of most of the exacerbations and the daily variability they present in the clinical manifestations of COPD may make it difficult for patients to recognise the beginning of an exacerbation. On reviewing the literature we did not find any study with a populational basis designed specifically to respond to the question regarding the incidence of exacerbations in patients attended in the community. In most studies the incidence is estimated from the follow up visits carried out at intervals of between 6 and 12 months. This may have repercussions on the quality of memory of the patients and thus, there may be a certain bias in memory so that only the most severe exacerbations requiring medical care or hospital admission are reported, while the self-limited exacerbations or those which reverse with the self-management of the patient remain unreported in epidemiological studies.

On the other hand, most studies are performed in populations attended in hospital services, with the consequent over-representation of moderate-severe COPD versus the milder forms of the disease. We therefore considered it necessary to carry out a multicentre study from primary care including the exacerbations of the less severe forms of the disease which are the most prevalent in the casuistry.

The study is therefore designed to better understand the epidemiology of the exacerbations of the less severe forms of COPD to know the incidence and the risk factors associated with exacerbations in the patients attended in primary care, including patients with mild involvement who have seldom been studied in previous investigations on exacerbations and COPD.

## Objectives

### Main objectives

1. To know the populational incidence of respiratory exacerbations in the COPD patients attended in the community.

2. To know the populational incidence of respiratory exacerbations in the COPD patients attended in the community based on the grade of disease severity.

3. To identify the risk factors associated with the appearance of exacerbations in COPD patients attended in the community.

### Secondary objectives

4. To know the proportion of patients incorrectly diagnosed with COPD in primary care offices.

5. To know the influence on the quality of life of COPD patients in relation to the number of exacerbations presented.

6. To know the degree of underdiagnosis of exacerbations in primary care offices.

7. To describe the healthcare resources used by COPD patients with an exacerbation.

## Methods and design

**Figure 1:**
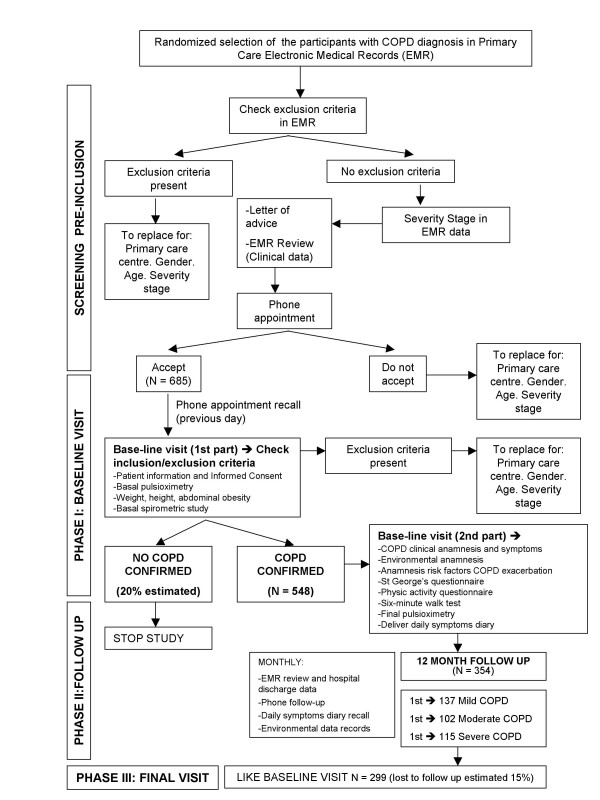
**Flow diagram of the study**.

A prospective, observational, populational, multicentre study will be carried out with a 12-month follow up divided into three phases (Figure 1): Phase I, initial evaluation with diagnostic confirmation and baseline evaluation of the study subjects; Phase II with a 12-month follow up to observe the appearance of episodes influencing an exacerbation and Phase III, the final evaluation.

### Study subjects

Patients who are smokers or ex-smokers diagnosed with COPD from 40 to 80 years of age without acute respiratory disease or any other long-term respiratory disease will be included. This multicentre study will include 21 primary care centres serving a population of 420,000 inhabitants in an urban, semirural and rural area of the zone of North Barcelona and the Maresme (Barcelona), Spain.

#### Inclusion criteria

Smokers or ex-smokers from 40 to 80 years of age diagnosed with COPD (Codes ICD-10: J43 and J44 or CIAP-2: R79 and R95) in the database of the computerised clinical history (e-CAP or OMI-AP) of the healthcare centres participating in the study without acute pulmonary disease at the time of inclusion and who accept to participate and sign the informed consent.

#### Exclusion criteria

Patients who have never smoked or patients with: contraindications for spirometry, difficulties in communicating (cognitive deterioration, sensorial disability, language barriers), severe disease with poor vital prognosis (less than one year), other active long-term respiratory disease during the last year (asthma, respiratory tract cancer, pulmonary thromboembolism, pulmonary tuberculosis, interstitial diseases, etc.), impossibility to communicate by telephone to establish the monthly follow up and patients who do not provide written informed consent to participate in the study.

Patients presenting acute respiratory disease on recruitment for Phase I will be invited to participate 30 days afterwards if the exacerbation has subsided. If another exacerbation is presented after one month the patient will be definitively excluded from the study. Acute respiratory disease is understood to be that presented in the 30 days prior to recruitment and includes: exacerbations of COPD, respiratory infection or hospital admission due to respiratory disease.

Phase I patients whose diagnosis of COPD is not confirmed on the basis of spirometric studies will be excluded from phases II and III.

### Sample size and selection method

All the calculations were carried out with an alpha risk of 0.05 and the follow up is fixed at one year. We believe that the incidence of exacerbations may be of around 1 per year, 2 per year and 4 per year in patients with mild, moderate and severe COPD, respectively. A total of 97 patients are necessary to estimate a rate of incidence of 1 exacerbation/person-year (with a precision of ± 0.2), 86 patients are required for a rate of 2 exacerbations/person-year (± 0.3) and 97 patients for a rate of 4 exacerbations/person-year (± 0.4). To detect a relative risk of 1.7 or more with a bilateral contrast with a proportion of subjects exposed to a certain risk factor between 20% and 70%, and guaranteeing a power of at least 80%, 116 patients are required if the rate of incidence of exacerbations between those not exposed is of 1/person-year, 58 patients if the rate is of 2 and 29 patients for a rate of 4. If the loss to follow up is estimated to be 15%, 137 mild patients, 102 moderates and 115 severe patients should be recruited to obtain the estimations of incidence and relative risk described. Assuming that 20% of the patients diagnosed with COPD in the clinical history are mild, 30% moderate, 30% severe and 20% are not really cases of COPD; to achieve a sample of 137 mild, 102 moderate and 115 severe patients a total of 685 patients should be recruited. With this sample can estimate a proportion of 20% of false diagnosis of COPD in a electronic medical records, with a precision of ± 0.3%. With 685 patients participating in Phase I only the first 137 mild patients, the first 102 moderate patients and the first 115 severe patients identified will be followed. With this sample a rate of weighted global incidence of between 1 and 4 exacerbations/person-year will be calculated with a precision of at least ± 0.4. A total of 801 exacerbations is expected. With this number of exacerbations a proportion of under-reporting of COPD exacerbations in the computerised clinical history of 20% may be estimated, with a precision of ± 0.3%.

The objective of the study is centred on the exacerbations of the less severe forms of COPD which are the most directly attended in primary care. This is why the severe and very severe classes of the stratification of severity of the Global Initiative for Chronic Obstructive Lung Disease (GOLD) [24] were included in the category of severe patients in the calculation of the sample.

The initial selection of the subjects will be performed randomly from the list of patients who are assigned one of the ICD-10 codes defining COPD, being stratified by grade of disease severity (according to data from the clinical history) and by the primary care centre. A letter will be sent to the subjects selected inviting them to participate in the study, with an appointment for the visit which will thereafter be confirmed by telephone.

### Study variables

#### Phase I, initial evaluation

The diagnosis and severity of COPD will be confirmed according to the GOLD criteria [24] performing a spirometry following a bronchodilation test in a stable setting. A diagnosis of COPD should be considered in any patients who has a post-bronchodilator FEV_1_/FVC ratio < 70%. The patients will be classified according to the severity of COPD as: mild (FEV_1 _≥ 80%), moderate (FEV_1 _≥ 50% and < 80%), severe (FEV_1 _≥ 30% and < 50%) and very severe (FEV_1 _< 30%) [24]. The following covariables will be collected in the subjects studied:

1. Baseline evaluation of the study subjects:

1.1 Oxygen saturation by pulsioximetry.

1.2 Usual symptomatology: grade of expectoration, presence of cough (usually no, occasionally or sometimes, everyday) and dyspnea with the MRC questionnaire [25].

1.3 Usual medication and treatment related to COPD: drugs and dosage.

1.4 Controls performed in relation to COPD: periodicity, healthcare level where carried out (family physician or pneumology specialist).

1.5 Previous hospital admissions in relation to COPD.

1.6 St. George's Respiratory Questionnaire (SGRQ) quality of life test [26].

1.7 Six-minute walk test [27].

2. Sociodemographic variables: age, gender, work status, level of education, municipality of residence, site of home (rural, semi-urban, urban) and ethnicity.

3. Physical activity usually undertaken, evaluated as weekly hours invested in: sedentary activities such as reading, watching television, manual activities, walking or strolling, going upstairs, or any other type of physical activity or sport.

4. Anthropometric variables: height, weight, waist and hip circumference.

5. Active and passive smoking exposure.

6. Labour and domestic exposure to possible risk factors of exacerbations: number of persons residing at home, presence of children or other chronic patients at home, presence of pets at home, environmental factors (dust, smoke, humidity, brusque temperature changes, air-conditioning, paint and solvents, combustible products, pollen).

7. Pathological antecedents and use of medications for other diseases. History of allergies. Vaccination status versus the influenza virus and pneumococci. The information reported by the patient will be compared with information from the clinical history.

#### Phase II, 12-month follow up

The variable of interest will be the appearance of an exacerbation of COPD. We define an exacerbation as any event in the natural development of the disease characterised by a change in baseline dyspnea, cough and/or expectoration of the patient beyond the daily variability and which is sufficient to justify a change in treatment [12]. A different episode of exacerbation will be considered when an interval of clinical stability of at least 72 hours is observed [28]. To determine the appearance of an exacerbation the patients will have a symptom diary at home and moreover they will be contacted by telephone every month.

They will be interrogated regarding changes in their usual symptomatology (expectoration, cough, dyspnea) as well as in the treatment for their respiratory disease. An increase in the symptomatology accompanied by a change in the treatment will be considered as a new event. To quantify the number of events in the last month indirect variables (number and type of consultation to healthcare services, hospital admissions due to COPD, changes in medication, evolution of the symptoms) will also be collected to know the delimitation of each episode. The patients will also be asked about the number of days they have felt worse than usual with respect to their disease, the number of times they have worsened and improved with respect to their disease during the last month and the healthcare resources used during each exacerbation. In addition, the appearance of respiratory morbidity (pneumonia, pulmonary neoplasm, heart failure, pulmonary embolism and pneumothorax) and death (date and cause) will be registered.

Each month the clinical histories of the primary care centres of all the patients followed will be examined, and when the patient has been hospitalised the clinical histories of the hospital of admission will be reviewed. With these data the grade of agreement between the information reported by the patient and that registered in the clinical history may be compared.

#### Phase III, final evaluation

The same variables as in the initial visit will be collected to detect changes in the quality of life, functional situation, symptoms, tolerance to effort, use of medication and use of healthcare resources.

### Data collection

#### Phase I

All the patients of the participating centres with a diagnosis of COPD encoded in their clinical history will be listed. A first screening will be made of these clinical histories from primary care and the hospital reports related to the same to exclude the patients with any criteria of exclusion in their clinical history or whose registered spirometry parameters are not congruent (FEV_1_/FVC ratio ≥ 70%) with a diagnosis of COPD. The patients who have not been excluded in this first screening will be randomly selected up to the number of patients required for each group of severity for each centre. The subjects selected will be invited to participate by letter and telephone call. If they do not wish to participate or cannot be located after 10 attempts to contact them on different days and times they will be substituted by another randomly chosen subject from the same centre. The patients selected who, at the time of study inclusion, have an exacerbation of their respiratory disease will be temporarily excluded and re-evaluated four weeks later. If the exacerbation situation persists at this time the patient will be definitively excluded from the study.

The patients selected will attend their healthcare centre without having smoked (minimum of 6 hours previously) or having taken their inhaled medication (12 or 24 hours previously according to the half life of the active drug). In the office they will be able to sign the informed consent to participate in the study. A nurse who is specifically trained for the study will perform a spirometry with a bronchodilator test. If the result of the spirometry is not compatible with a diagnosis of COPD, the study will be finished.

If the result of the spirometry is compatible with the diagnosis of COPD the tests, measurements and questionnaire described in the section of study variables will be performed up to completing the groups of 137 mild patients, 102 moderate and 115 severe patients necessary for the follow up. Since this Phase I cannot be carried out simultaneously in all the centres participating in the study to ensure that the patients remain similarly distributed in number and severity in the different centres, each centre will be proportionally assigned the patients of each group of COPD severity.

The information will be introduced by the nurse in a database during the visit using an electronically supported case report form (CRF) and portable computer.

#### Phase II

The first 137 mild patients, 102 moderate and 115 severe patients detected in

Phase I will participate in the second phase of the study.

One month after the patient in Phase I has been included, a nurse especially trained for the study will perform the telephone follow up with the standardised questions which will allow collection of the variables described in the section on study variables to detect the appearance of exacerbations in this period of time. If the patient is admitted or is unable to respond to the telephone questionnaire the data will be obtained from the main patient care provider. If the patient cannot be localised after 10 attempts, the questionnaire will be postponed until the following month. The telephone follow up will be carried out monthly in all the patients included in Phase II of the study. Data collection will also be undertaken with an electronically supported CRF and a portable computer at the same time as the telephone follow up.

Each patient included in Phase II will analogously receive a diary edited specifically for the study to note the changes in the symptoms of the disease and/or treatment and the need to use healthcare service due to an exacerbation daily. This diary is designed graphically thereby helping the patients to carry out this task. Each page of the diary collects the information corresponding to one month and may be taken out so that the patients may hand in the registry sheet to their primary healthcare centre. If necessary, the centre may help to solve any doubts regarding this note taking, although the follow up telephone call may also be helpful or the patients may call a telephone number given to all the patients included in the study.

#### Phase III

The week before completing one year of inclusion in the study, the patients who have participated in Phase II will be contacted to be invited to participate in Phase III. The same tests, measurements and questionnaires performed at the beginning of the study (Phase I) will be performed and the patient will be interrogated regarding the appearance of exacerbations during the last month. Assuming that 15% of the patients will be lost to follow up, it is estimated that 116 mild patients, 86 moderate and 97 severe patients, according to the initial evaluation of severity, will participate in this part of the study.

Prior to definitively launching the study, a pilot test will be undertaken with 50 subjects for the Phase I and the first month of follow up. Following analysis of this test the processes which have not worked satisfactorily will be redesigned.

The period of inclusion of the patients in Phase I is estimated to be of 10 – 12 months, the follow up in Phase II will be of 12 months and Phase III will be completed in 4–6 months more. Thus, the foreseen duration of the study is 30 months. At the end of 2009 Phase I results will be presented and the conclusions and final results of the study will be available in 2011.

### Analysis plan

To respond to objectives 1 and 2 the rate of incidence will be estimated as the number of exacerbations per patient-year. To do this, negative binomial regression models will be used to calculate the incidences and their confidence intervals [29]. The relative risks (RR) of incidence between individuals exposed and not exposed to a certain risk factor will be used to respond to the third objective [29]. With these models the changes observed between the baseline and final situation will be related as will the number of exacerbations (objective 5). To determine the RR of the different variables, bivariate and multivariate models will be applied adjusting for the potential factors of confusion. All the analyses will be globally performed, being weighted for the distribution of severity obtained in Phase I and according to the severity of the patient. Descriptive statistics will be carried out of all the variables collected (objective 7). The number of patients whose diagnosis of COPD is not confirmed in Phase I, divided by the total number of patients visited in this phase, will provide the proportion of patients with an incorrect diagnosis of COPD in primary care (objective 4). The number of exacerbations not collected in the clinical histories from primary care, divided by the number of exacerbations reported by the patient in Phase II of follow up, will provide the grade of under reporting of the exacerbations in primary care (objective 6). All the statistical tests will be carried out with a bilateral confidence level of 95%.

### Ethical considerations

Written informed consent will be requested from all the patients prior to their inclusion in the study. The patients will be informed of the objectives of the study as well as the tests and the follow up to be carried out.

This study has been approved by the Ethical Committee of Clinical Investigation of the Primary Care Research Institute Jordi Gol (Barcelona, Spain).

## Discussion

The results of the study will provide knowledge regarding aspects of COPD which are little known, such as the natural history of the initial phases of the disease and the impact and incidence of the exacerbations on the patients with mild-moderate forms of the disease attended at a primary healthcare level. Most of the current knowledge of this disease is focused on its most severe forms, in patients mainly attended in hospital centres, who are possible only the "tip of the iceberg" of a much deeper problem. We also expect to provide results related to the incidence and impact on health of these mild-moderate recurrences, which, in many cases, are not attended in the healthcare system until they acquire criteria of severity. This knowledge will allow early intervention strategies for the recurrences to be designed, promoting self-care and the early identification of the symptoms as basic elements to minimise their progression to severity and the use of more complex healthcare resources. We will detect possible variations in the seasonal incidence of exacerbations in our setting along with their implications for the planning of service availability and healthcare resources in the months of highest incidence. Likewise, we expect to obtain more in depth knowledge of the risk factors which favour the exacerbation of COPD, with their implications to provide schedules to avoid the exposure to these factors.

We believe it will be relevant to provide data related to the milder forms of exacerbation which are often silent or very little expressed clinically and which are not usually seen in the healthcare system. These data may be particularly important in patients with less severe forms of COPD who, given the chronicity and progressiveness of this disease, will, in the near future, be among the 5 first cause of morbimortality in western countries.

One important limitation of this study is the definition of exacerbation itself and the ability to differentiate the exacerbations of COPD from other acute respiratory processes in patients with the disease. This difficulty will be minimised using the definition adopted by the consensus between the European Respiratory Society and the American Thoracic Society. This definition also establishes a minimum period of 72 hours of clinical stability between one exacerbation and the next. Thus, performing a monthly follow up will ensure better patient memory with respect to the exacerbations experienced, thereby reducing the risk of introducing a memory bias on behalf of the patients.

At the same time, to carry out a more exhaustive follow up of the episodes, the clinical histories of the patients will be reviewed at both an outpatient and hospital level. Moreover, the patients will be able to personally note down the changes in their disease and/or treatment in the diary specifically edited for the study. The results will be compared with the data from the 3 sources of follow up information (medical registries, telephone call and symptom diary).

From the experience accumulated in previous studies we expect the rate of participation in the study to be high, particularly if we take into account the implication of the professionals of the primary healthcare centres of the patients. On the other hand, since this is an observational study which does not involve the performance of invasive tests or pharmacologic interventions, we believe this will also facilitate recruitment and patient inclusion.

To increase the validity and reliability of the diagnostic tests performed, the collaborating personnel of the study will be specially trained to perform these tests and the same spirometry model will be used in all the patients.

In conclusion, we believe that the results of the study will provide knowledge to know the characteristics of the patients with COPD attended in primary care better and will help to achieve the recommendations of the management of the disease contained in the national and international guidelines [24,30].

## Abbreviations

CIAP: International Classification of Primary Care, Spanish version; COPD: Chronic Obstructive Pulmonary Disease; CRF: Case report form; FEV_1_: Forced Expiratory Volume in the first second; FVC: Forced Vital Capacity; GOLD: Global Initiative for Chronic Obstructive Lung Disease; ICD: International Classification of Diseases; MRC: Medical Research Council; RR: Relative Risk.

## Competing interests

The authors declare that they have no competing interests.

## Authors' contributions

EB, MR and PT contributed to the original research idea about the incidence of COPD exacerbations in primary healthcare centres. EB, MR, PT, LM, GP, NM and MM participated in the design of the study. GP and LM participated in the statistical analysis and in the design of research protocol. AN, MU, YP, XM, CD, AL, MLG, SM, GT, contributed to the coordination of the study and technical support in spirometric tests. All the authors have read, revised and approved the final manuscript.

## Pre-publication history

The pre-publication history for this paper can be accessed here:


